# The Breadth of Black

**Published:** 1871-06

**Authors:** 


### THE BREADTH OF BLACK,

As seen at the ends of the fingers under the nails, is the measure of civilization, the measure of an unrefined mind. The broader the rim, and the blacker, the more contemptuously do we look on the wearer. There are three ways of removing it; a very common method, a kind of pastime with some, is to scrape it out with the point of a penknife, and wipe it off on the pantaloons; another method is practiced by school-girls, who eat it off, in their habit of nibbling at the nails; a third, and the only proper plan, is to keep the finger-ends well washed. If a penknife is used, violence is offered to the tender surface under the nails; the skin is made callous, and sometimes ugly sores make their appearance. The nails should never be cut to the quick, because they were intended to protect the ends of the fingers, helping to preserve the nicety of touch, so essential to the needs of life; a little over the sixteenth of an inch, or not quite the thickness of a nickel cent, is an abundant margin. The nails should be trimmed with toilet scissors, and never with a knife, unless done with great care and deliberation, so as to avoid slips, which might injure the nail or cause troublesome bleedings.

This black accumulation under the finger-nails is a mixture of oil and dust, and is the essence of filthiness; a clean hand is the only proper remedy; if eaten off as above stated, or scraped away, the hand remains unclean.

The nails should be pared on fixed days, once or twice a week; they should never be scraped, as thereby they are thickened, and made to have white splotches if the scraping is done where the skin joins on to the nail, which are quite a disfigurement.

Sometimes very troublesome sores are occasioned where the nail comes out from the skin; this is caused by the skin growing to the nail, and the nail, growing or pushing out from the hand, drags the skin along with it, first distending and then snapping it, when the ends of the skin stick upward; meanwhile such a degree of inflammation is induced that a painful soreness is occasioned, which does not get well for a week or ten days.

Some persons loosen this skin from time to time with the point of a penknife, moving the knife from side to side under the edge of the skin; this scraping occasions the white spots sometimes seen on the nail, which are very unsightly; the best way to loosen the skin is to do it with the thumb-nail of the other hand, pushing it up, or with an ivory paper-cutter, or handle of a tea-spoon, all these being blunt; another way is, every time the hands are washed, push the towel on the finger-nails towards the body; this prevents the adhesion of the skin to the nails.

